# Vitamin D receptor polymorphism rs2228570 is significantly associated with risk of dyslipidemia and serum LDL levels in Chinese Han population

**DOI:** 10.1186/s12944-018-0819-0

**Published:** 2018-08-17

**Authors:** Jian Jia, Yayu Tang, Chong Shen, Ning Zhang, Haixia Ding, Yiyang Zhan

**Affiliations:** 1 General Internal Medicine Department, The First Affiliated Hospital of Nanjing Medical University, Nanjing, China; 2 Geriatric Medicine Department, The First Affiliated Hospital of Nanjing Medical University, Nanjing, China; 3 Department of Epidemiology and Biostatistics, School of Public Health, Nanjing Medical University, Nanjing City, Jiangsu Province China

**Keywords:** Vitamin D receptor, Polymorphism, Dyslipidemia, Chinese

## Abstract

**Background:**

The goal of this study was to determine if vitamin D receptor (VDR) gene polymorphisms underlie susceptibility to dyslipidemia in a Chinese Han population.

**Methods:**

Three tag single nucleotide polymorphisms (SNPs) (rs11574129, rs2228570, and rs739837) were genotyped using TaqMan assays to determine *VDR* SNP associations with dyslipidemia. We genotyped 877 cases of dyslipidemia from a normotensive, non-diabetes mellitus population and 1822 non-dyslipidemia subjects in a stage I study. In a follow-up stage II study, we included a larger sample of 3124 controls and 1679 cases with dyslipidemia. Finally, we explored the potential molecular mechanism for the SNP associations using molecular modeling analysis.

**Results:**

We found a significant association between SNP rs2228570 and dyslipidemia in the additive (adjusted odds ratio (OR) = 1.255, 95% Confidence Interval (CI) = (1.118–1.409), *P* < 0.001), dominant (OR = 1.384, 95% CI = 1.384 (1.136–1.6), *P =* 0.001) and recessive models (OR = 1.356, 95%CI = 1.1–1.671, *P =* 0.004) in stage I. We further established that the rs2228570 variant was significantly associated with dyslipidemia in the additive (adjusted OR = 1.146, 95% CI = 1.053–1247, *P* = 0.002), dominant (OR = 1.184, 95%CI =1.018–1.376, *P =* 0.028) and recessive models (OR = 1.209, 95%CI = 1.064–1.374, *P =* 0.004) in stage II. The TT genotype was significantly higher (4.93 ± 0.75 mmol/L) compared to the TC (4.67 ± 0.47 mmol/L) or CC (4.66 ± 0.44 mmol/L) genotype (*P* = 0.01) in cases with elevated low-density lipoprotein cholesterol (LDL-C) levels. In contrast, the cases with the TT genotype had significantly lower serum 25(OH)D levels (18.43 ± 5.04 ng/ mL) compared to the TC (26.24 ± 4.16 ng/mL) and CC (36.76 ± 8.10 ng/ mL) genotypes (*P* < 0.001). Multivariable linear regression analysis indicated that the rs2228750 genotype significantly correlated with serum low-density lipoprotein-C (LDL-C) levels in cases with dyslipidemia. Using molecular modeling analysis, we further found that the rs2228570 variant changed the structure and the stability of VDR and altered the binding energy of its ligand.

**Conclusions:**

The *VDR* rs2228570 variant may increase susceptibility to dyslipidemia in the Chinese Han population.

**Electronic supplementary material:**

The online version of this article (10.1186/s12944-018-0819-0) contains supplementary material, which is available to authorized users.

## Background

The prevalence of lipid abnormalities is increasing in the Chinese Han population, and is considered as a major risk factor for cardiovascular disease. Low vitamin D status has been reported to be correlated with an increased risk of hyperlipidemia [1, 2]. Furthermore, a correlation was found between vitamin D deficiency and increased serum total cholesterol (TC) and low-density lipoprotein-cholesterol (LDL-C) levels, and decreased serum high-density lipoprotein-C (HDL-C) levels [3–5]. Nonetheless, the mechanism of the lipid-lowering effect of vitamin D remains unknown. Vitamin D likely increases serum calcium by enhancing intestinal calcium absorption. Subsequently, this elevated calcium level reduces serum triglycerides by suppression of hepatic triglyceride formation and secretion [6]. The second possible mechanism may be through the inhibitory effect of vitamin D on serum PTH concentrations. The lowered level of PTH may reduce serum triglycerides by increasing their peripheral uptake. Another potential mechanism to explain the association between 25(OH) D and triglycerides would be through insulin resistance. In cases of vitamin D deficiency, the risk of insulin resistance increases, which is associated with elevated levels of very-low-density lipoprotein (VLDL) cholesterol and triglycerides [7].

Dyslipidemia is a multifactorial disease, which can be influenced by both genetic and environmental factors. Vitamin D is involved in lipid metabolism. The actions of vitamin D are mediated by the Vitamin D Receptor (VDR), a nuclear receptor [8]. The *VDR* gene, which codes for VDR, is located at q11–q13 on chromosome 12. A recent study showed that the CC genotype of *VDR,* single nucleotide polymorphism (SNP) rs2228570, is a risk factor for diabetic dyslipidemia in elderly males in North China [9]. Another investigation, conducted in Lebanon, suggested that *VDR* polymorphisms were associated with triglyceride and HDL levels in a healthy young population [10]. VDR FokI polymorphisms appear to be associated with coronary heart disease in Han Chinese. Furthermore, the GG genotype predicts higher HDL-cholesterol levels in adults with coronary heart disease [11]. Considering the lack of studies evaluating the link between *VDR* and dyslipidemia, we investigated and determined the association between *VDR* variants and dyslipidemia in a large Chinese Han sample.

## Methods

### Ethics Statement

The study was approved by the Ethics Committees of the First Affiliated Hospital with Nanjing Medical University, Nanjing, Jiangsu, China (NO2015-SR-032). All participants signed written informed consent.

### Subjects

A two-stage designed study was used to evaluate whether genetic variations in *VDR* are associated with dyslipidemia in the Chinese Han population. In stage I, we genotyped three SNPs (rs11574129, rs2228570, and rs739837) in 877 cases with dyslipidemia from normotensive and non-diabetes mellitus population and 1822 non-dyslipidemia subjects from a community-based epidemiological survey in Jiangsu Province, China. We followed-up the stage I study to confirm the association between rs2228750 and dyslipidemia using a larger sample, including 1679 cases with dyslipidemia and 3124 age-, sex-, and ethnic origin-matched controls, in a stage II study.

Dyslipidemia was identified according to the Guidelines on Prevention and Treatment of Dyslipidemia in Chinese Adults (2007) [12]: triglyceride (TG) ≥ 2.26 mmol/L as high, total cholesterol (TC) ≥ 6.22 mmol/L as high, low-density lipoprotein cholesterol (LDL-C) ≥ 4.14 mmol/L as high, and high-density lipoprotein cholesterol (HDL-C) < 1.04 mmol/L as low. In our study, high TG, high TC, high LDL-C, or low HDL-C was regarded as dyslipidemia. The exclusion criteria included a history of cancer, stoke, thyroid disorder, kidney, or liver disease. Individuals who had family history of obesity, dyslipidemia, and other metabolic diseases were also excluded. All individuals answered standardized questionnaires inquiring about age, sex, ethnicity, history of smoking, and family medical history. Body-mass index (BMI) was calculated as weight divided by height squared in kg/m^2^. Blood pressure was measured using a mercury sphygmomanometer. LDL-C, HDL-C, TC, TG, and fasting blood glucose (GLU) serum levels were measured enzymatically using an automatic clinical analyzer (Hitachi Inc., Tokyo, Japan). Serum 25(OH) D concentration was determined using an enzyme-linked immunosorbent assay (ELISA) according to the product specifications (Eagle Biosciences Inc., Nashua, New Hampshire, USA).

### Genomic DNA extraction and genotyping

Genomic deoxyribonucleic acid (DNA) was extracted from whole-blood samples using the TIANGEN RelaxGene Blood DNA System (TianGen Biotech Co, Lid, Beijing, China; NO. DP319). Three tagSNPs (rs11574129, rs2228570, and rs739837) were selected using the linkage disequilibrium (LD) method and SNP function prediction software (https://manticore.niehs.nih.gov/snpinfo/snpfunc.html). The rs11574129 and rs739837 variants regulating the microRNA binding were positioned in the 3’untranslated region. The rs2228570 was predicted as a splicing enhancer in the exon-coding region. Genotyping was performed using the TaqMan SNP Genotyping Assay (Applied Biosystems, Foster City, CA, USA). For quality control, 5% of samples were re-genotyped in a blinded fashion.

### Molecular Modeling Analysis

The *VDR* molecular modeling studies using Discovery Studio 3.0 (DS 3.0, Accelrys Inc., Boston, MA, USA) provided insight into two *VDR* isoforms (wild type and RS2228570 variant). These *VDR* isoforms were produced by I-TASSER (http://zhanglab.ccmb.med.umich.edu/I-TASSER/). We selected the most appropriate structure from five models to model *VDR* by FG-MD (http://zhanglab.ccmb.med.umich.edu /FG-MD/). Then, we used DS 3.0 to set minimization (steepest descent, conjugate gradient) and equilibration steps, followed by CHARMM to understand the molecular dynamics.

### Statistical analysis

Data analyses were performed using SPSS 19.0 (SPSS, Inc., Chicago, IL, USA). Quantitative variables were expressed as mean ± standard deviation (SD). Comparisons of the means between the two groups were determined by *t*-test, and the multiple-group means were compared using one-way ANOVA, with adjustments for age and sex. The Chi-square (χ^2^) test or Fisher’s exact test was employed for categorical variables. Multiple logistic regression analysis was applied to adjust covariates including age and sex. In addition, multivariable linear regression was utilized to evaluate the association of genotypes with lipid levels. Hardy–Weinberg equilibrium (HWE) was assessed by Fisher’s exact χ^2^ test in the control. Statistical significance was assumed at *P* < 0.05.

## Results

### Study subject characteristics

A total of 436 males and 441 females were included in the case group and 926 males and 956 females were included in the control group in the stage I study. The average levels of BMI, LDL, and TC in the case group were significantly higher than those in the controls. Compared to controls, the case groups had a significantly lower level of HDL. However, there was no significant difference in systolic blood pressure (SBP), diastolic blood pressure (DBP), gender, age, and GLU between the case and control groups (Additional file 1: Table S1).

The clinical characteristics of the cases the in stage II study are presented in Additional file 1: Table S2. The case group consisted of 834 males and 845 females, whereas 1515 males and 1609 females were included in the control group. We observed no significant differences in gender and age between the case and control groups. However, the groups differed significantly in their mean BMI, SBP, DBP, GLU, TC, TG, LDL-C, and HDL-C levels. The hypertension prevalence in the case group was significantly higher than that in the control group.

### Association of VDR polymorphisms with dyslipidemia

In the present study, the *VDR* genotype (rs11574129, rs2228570, and rs739837) frequencies were in the Hardy-Weinberg equilibrium (both *P* > 0.05). In the stage I study, we found that the SNP rs2228570 statistically correlated with an increased risk of dyslipidemia; after adjusting for confounding factors, the odds ratios (ORs) with 95% confidence interval (CI) of the additive, dominant, and recessive models were 1.255 (1.118–1.409) (*P* < 0.001),1.384 (1.136–1.6)(*P* = 0.001) and 1.356 (1.1–1.671)(*P* = 0.004) (Table 1).

**Table 1:** Association analysis of the three *VDR* SNPs and cases (stage I)

SNP	Group	Genotype OR (95% CI)^a^					Allele		
		WT	Ht+ MT	Additive	Dominant	Recessive	Major/Minor	OR (95% CI)	*P* -value
rs11574129		TT	TC + CC				T/C		
	Control	1265	560 + 55	1.069 (0.923–1.238)	1.055 (0.89–1.25)	1.2 6 (0.809–1.964)	3090/670	1.07 (0.92–1.24)	0.46^c^
	case	578	265 + 32	*P* = 0.376	*P* = 0.536	*P* = 0.306	1421/329		0.38^b^
rs2228570		CC	TC + TT				C/T		
	Control	539	939 + 401	1.255 (1.118–1.409)	1.384 (1.136–1.6)	1.356 (1.1–1.671)	2017/1741	1.25 (1.12-1.41)	0.832^c^
	case	308	422 + 146	*P* < 0.001	*P* =0.001	P = 0.004	1038/714		0.001^b^
rs739837		CC	TC + TT				C/T		
	Control	991	749 + 139	1.094 (0.965–1.241)	1.108 (0.944–1.301)	1.157 (0.862–1.553)	2731/1027	1.09 (0.96–1.24)	0.877^c^
	case	439	362 + 74	*P* =0.161	*P* = 0.209	*P* = 0.33	1240/510		0.17^b^

^a^ Adjusted for sex and age

^b^ *P-*value of χ^2^ test for comparison of allele between case and control group

^c^ *P-*value of Fisher’s exact χ^2^ test for HWE

WT wild type, Ht heterozygote, MT mutant type

OR Odds ratio

CI Confidence interval

Based on the results of the stage I study, we focused on the relationship between the rs2228570 variant and the risk of dyslipidemia in the stage II study. Subsequently, we conducted a second-stage replication by genotyping a larger case-control population. We found that SNP rs2228570 significantly correlated with an increased risk of dyslipidemia; after adjusting for confounding factors, the ORs with 95% CI of the additive, dominant and recessive models were 1.146 (1.053–1247) (*P =* 0.002), 1.184(1.018–1.376)(*P =* 0.028) and 1.209 (1.064–1.374)(*P =* 0.004) (Table 2). To reduce potential bias in the stage II study, we adjusted for confounding factors, such as blood pressure, smoking, BMI, and GLU. In the GLU-adjusted model, we found a significant association between the rs2228570 variant and increased risk of dyslipidemia. After adjusting for confounding factors, the ORs with 95% CI of the additive, dominant, and recessive models were 1.145 (1.052–1.246) (*P* = 0.002), 1.177 (1.013–1.369) (*P* = 0.034), and 1.211(1.065–1.377) (*P* = 0.003). A similar trend was observed in the blood pressure-, smoking-, and BMI-adjusted models (Table 3).

**Table 2:** Association analysis of the rs2228570 and cases (stage II)

SNP	Group	Genotype OR (95% CI)^a^					Allele		
		WT	Ht + MT	Additive	Dominant	Recessive	Major/Minor	OR (95% CI)	*P-*value
rs2228570		CC	TC + TT				C/T		
	Control	902	1558+661	1.146 (1.053–1,247)	1.184 (1.018–1.376)	1.209 (1.064–1.374)	3362/2880	1.15 (1.05-1.25)	0.806^c^
	case	553	815 + 310	*P* = 0.002	*P* = 0.028	P = 0.004	1921/1435		0.002^b^

^a^ Adjusted for sex and age

^b^ *P-*value of χ^2^ test for comparison of allele between case and control group

^c^ *P-*value of Fisher’s exact χ^2^ test for HWE

WT wild type, Ht heterozygote, MT mutant type

OR Odds ratio

CI Confidence interval

**Table 3:** Stratified analysis of the association of rs2228570 and dyslipidemia

Genotype	Genotype OR (95% CI)			
	OR (95% CI)^a^	OR (95% CI)^b^	OR (95% CI)^c^	OR (95% CI)^d^
Additive	1.145 (1.052–1.246)	1.145 (1.052–1.247)	1.144 (1.051–1.246)	1.142 (1.047–1.244)
	*P* = 0.002	*P* = 0.002	*P* = 0.002	***P*** **= 0.003**
Dominant	1.177 (1.013–1.369)	1.188 (1.021–1.381)	1.181 (1.016–1.373)	1.18 (1.013–1.375)
	*P* = 0.034	*P* = 0.026	*P* = 0.03	*P* = 0.034
Recessive	1.211 (1.065–1.377)	1.204 (1.059–1.369)	1.207 (1.062–1.371)	1.201 (1.054–1.368)
	*P* = 0.003	*P* = 0.005	*P* = 0.004	*P* = 0.006

^a^ GLU adjusted model

^b^ SBP and DBP adjusted model

^c^ Smoking adjusted model

^d^ BMI adjusted model

OR Odds ratio

CI Confidence Interval

**Fig. 1:**
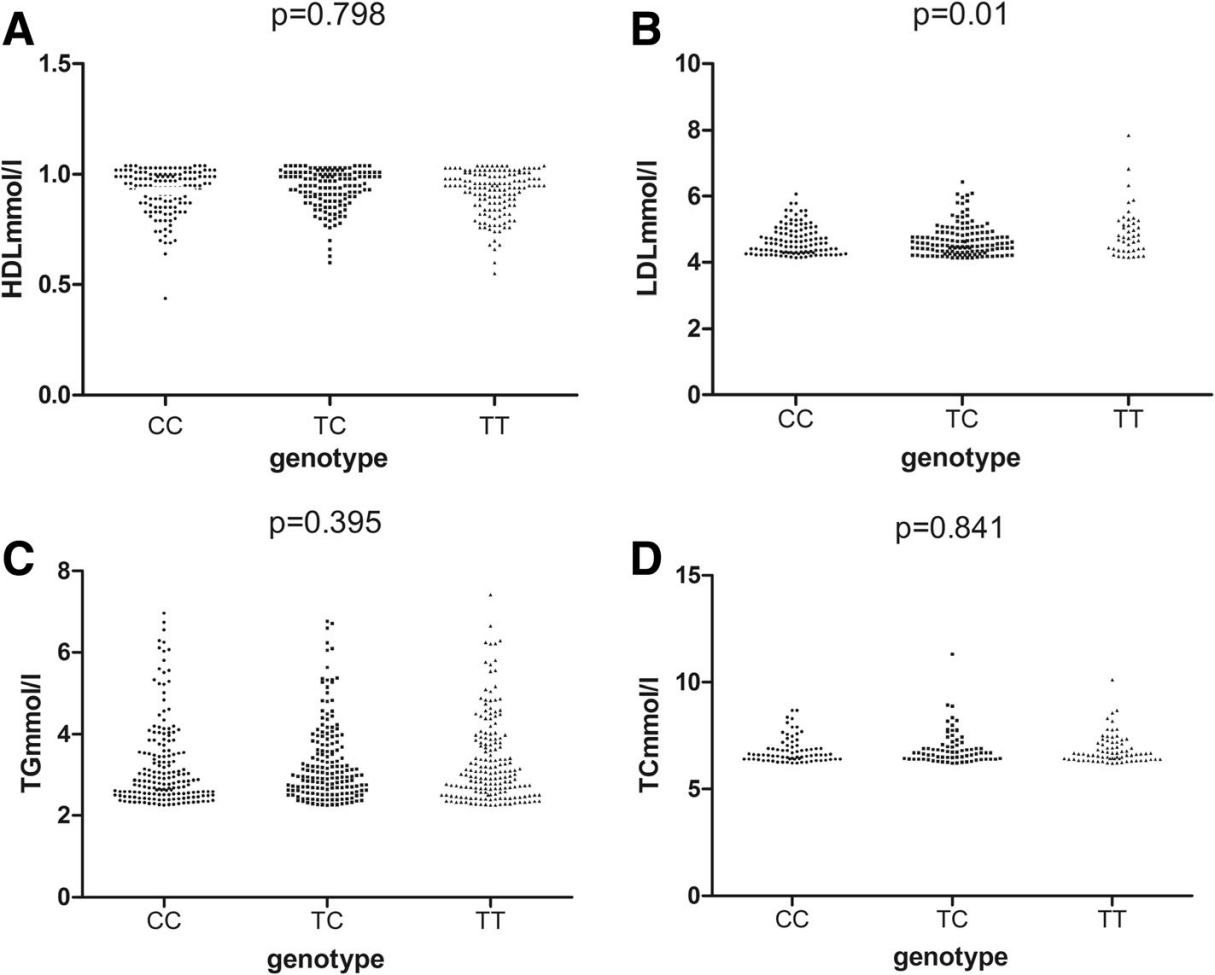
Serum lipid levels in the different genotypes cases. **a** Levels of HDL distribution in the three genotype cases; **b** Levels of LDL distribution in the three genotypes cases; **c** Levels of TG distribution in the three genotypes cases; **d** Levels of TC distribution in the three genotypes cases. The values are means ± SD

**Fig. 2:**
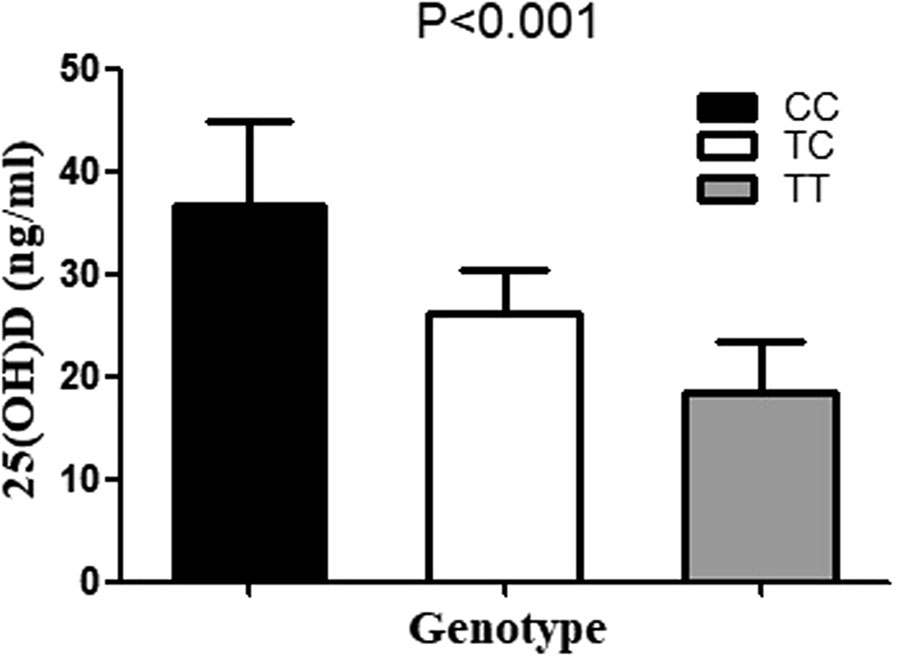
Serum 25(OH)D levels in the different genotypes in the cases with high LDL levels. The three bars indicate serum 25(OH)D distribution in the three genotype cases. The values represent means ± SD

Serum lipid levels in the cases with different genotypes are shown in Fig. 1, in which it can be seen that the rs2228750 genotype was significantly associated with serum LDL levels in the dyslipidemia cases. The cases with the TT genotype had significantly higher LDL levels (4.93 ± 0.75 mmol/L) than those with the TC (4.67 ± 0.47 mmol/L) or CC (4.66 ± 0.44 mmol/L) genotype (*P* = 0.01). There were no significant differences among the three genotypes in the levels of serum TC, TG, or HDL (*P* > 0.05) (Additional file 1: Table S3). The levels of serum 25(OH)D among the different genotype cases with high LDL levels (≥ 4.41 mmol/L) is illustrated in Fig. 2. In the cases with high LDL, the cases with the TT genotype had significantly lower serum 25(OH)D levels (18.43 ± 5.04 ng/mL) than those with the TC (26.24 ± 4.16 ng/mL) or CC (36.76 ± 8.10 ng/mL) genotype (*P* < 0.001) (Additional file 1: Table S4).

Multiple linear regression analysis revealed that the rs2228570 variant (*P* = 0.041), age (*P* = 0.01), and sex (*P* = 0.021) were independently related to the level of serum LDL (Table 4).

**Table 4:** Linear regression analysis of LDL levels

Variable	Beta	B (95% CI)	*P*
rs2228570	-0.117	-0.089 (0.173–0.004)	0.041
Age	0.152	0.007 (0.002–0.012)	0.01
Sex	0.141	0.146 (0.022–0.27)	0.021
GLU	0.09	0.065 (-0.019–0.149)	0.131
BMI	0.096	0.017 (-0.003–0.038)	0.365
Smoking	0.129	0.169 (0.014–0.325)	0.053

GLU, fasting blood glucose; BMI, body mass index

### Molecular modeling study

**Fig. 3:**
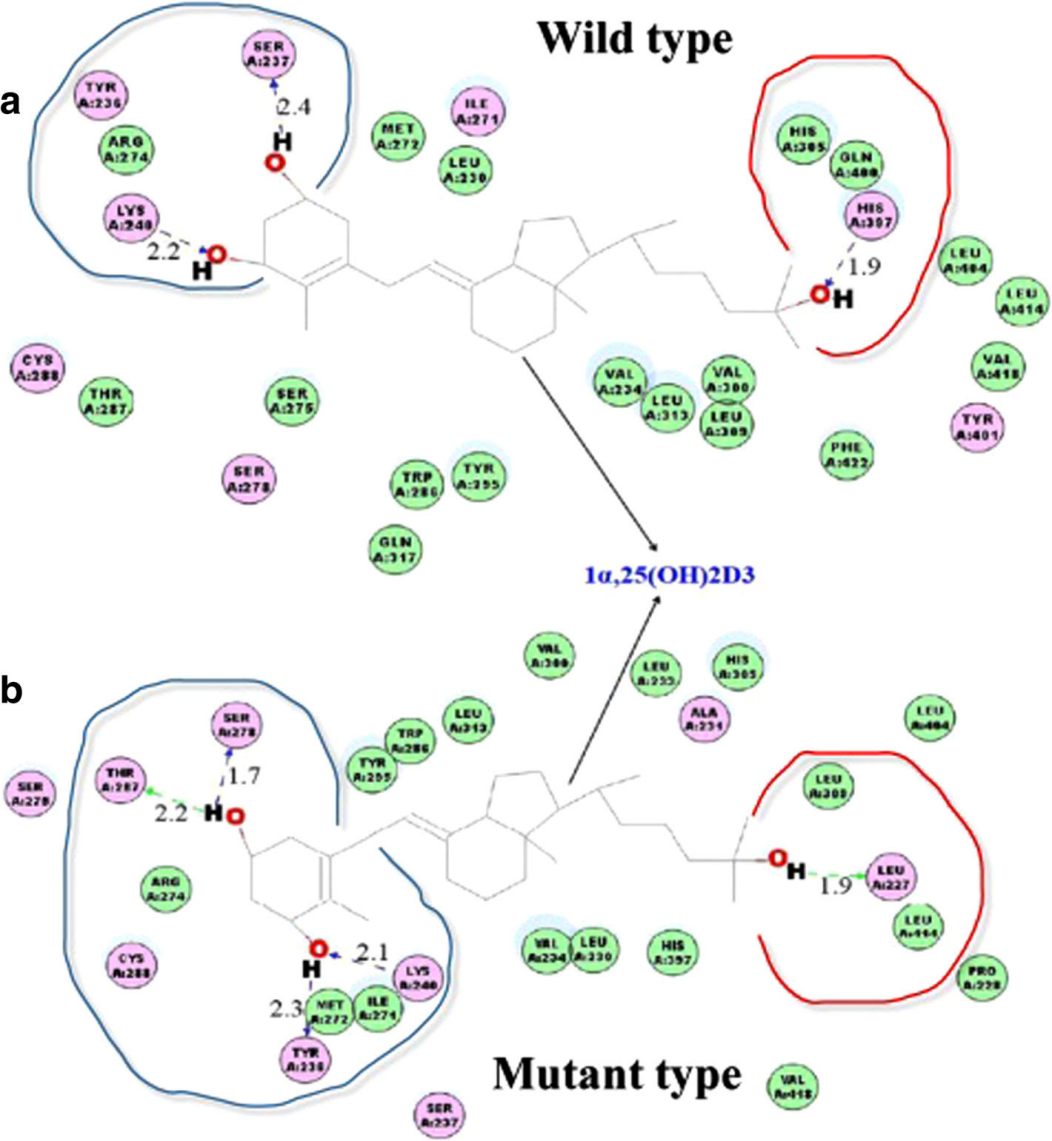
2D diagram of the changes in bound ligand conformations of the wild-type and mutant VDR. The superposition of 1,25(OH)2D3 molecules is shown in gray: Circles represent the amino acids of the receptor molecule. Blue halos around the circles denote the solvent-accessible surface of the interacting residue. The dashed lines indicate the hydrogen-bond interactions between the ligands and the receptor molecule. The green dashed lines represent hydrogen bond interactions with amino acid main chains; blue-dashed lines show the hydrogen bond interactions with the main chains of the amino acids (the arrow head is directed towards the electron donor). The numbers (e.g., 2.2) represent the hydrogen bond interaction distances: **a** Conformation derived from the VDR receptor wild-type model structure; **b** Conformation derived from the VDR receptor mutant model structure (MET1THR)

The molecular modeling analysis showed that VDR protein stability was reduced in the rs2228570 variant. The binding energy of VDR to its ligand was also decreased in the rs2228570 variant (Additional file 1: Tables S5, S6). As can be observed in Fig. 3, the conformation and interaction changes are in the polymorphic region (rs2228570), where the replacement of threonine with methionine at amino acid position 1 changed the structure of VDR. This modification resulted in a shift of the mode in which the amino acid residue Tyr236 in the protein VDR was bound to hydrogen in its ligand. The change also provided interactions with neighboring residues.

## Discussion

The active form of vitamin D is 1, 25-dihydroxyvitamin D. VDR tightly regulates vitamin D levels in cells and in the circulation [13]. A previous study [14] revealed that VDR activation repressed the increased levels of mouse and human CYP7A1 and reduced the cholesterol concentrations exerted by hepatic small heterodimer partner (SHP). Recent research found that the deletion of macrophage VDR promoted insulin resistance and monocyte cholesterol transport, which accelerated atherosclerosis in mice [15]. Based on the findings of the abovementioned studies, we hypothesized that VDR expression is associated with serum lipid levels. Hence, further studies on *VDR* polymorphisms would improve the understanding of the association of VDR with dyslipidemia.

In our study, we found a significant association of rs2228570 polymorphism with dyslipidemia and serum LDL levels. Moreover, cases with the TT genotype had significantly lower serum 25(OH)D levels compared to cases with other genotypes with high LDL.

As is well known, vitamin D regulates lipid metabolism; it inhibits lipid synthesis and promotes adipopexis in adipocytes [16]. However, the underlying mechanisms remain largely unknown. It is possible that the potential underlying mechanism operates through facilitating calcium homeostasis via vitamin D. It is commonly known that vitamin D and parathyroid hormone (PTH) are the two most important hormones that maintain calcium levels [17, 18]. Therefore, the reduction in lipid concentrations observed in this study may have been caused by increased calcium levels due to a decrease in fatty acid absorption and an increase in fecal fatty acid content, probably arising from the formation of insoluble calcium-fatty soaps in the gut [19, 20]. Such reduced absorption of fat diminishes serum total and LDL cholesterol levels [21, 22]. Calcium can also bind bile acid [23, 24] and increase the conversion of cholesterol to bile acids, thus causing increased cholesterol excretion [25]. Furthermore, microsomal triglyceride transfer protein (MTP) is involved in the secretion of triglyceride-rich VLDL, which leads to hypertriglyceridemia and atherosclerosis [26]. In addition, hepatocellular calcium activates MTP. The increase in hepatocellular calcium could be restrained by increasing serum concentrations [23]. The inhibition of hypertriglyceridemia may be caused by the inhibition of MTP activity via the suppression of hepatocellular calcium. Another potential mechanism is likely to be associated with a decrease in cholesterol absorption [27]. Vitamin D increases intestinal calcium absorption by affecting the rate of fat absorption, which results from the ability of calcium to bind to fatty and bile acids, forming calcium-fatty acid soaps [27], which are excreted in the feces. Moreover, vitamin D may play a role in insulin secretion and sensitivity, thereby indirectly influencing lipid metabolism [28].

Using our molecular model, we further established that rs2228570 changed the structure and stability of VDR and altered the binding energy of its ligand. Earlier investigations revealed that an association between low levels of vitamin D and rs2228570 located in *VDR’s* 5’end. rs2228570 has a T to C variation in the translation initiation codon (AGT) in exon 2 [29]. This change induces the synthesis of a small (49.5 kD) protein with increased biological activity [30]. The reason for the differences between the activities of the two proteins, such as their abilities to bind 1,25-dihydroxy vitamin D_3_ or induce transcription, still remain to be determined.

## Conclusions

In conclusion, our results suggest that the rs2228570 polymorphism correlates with an increased risk of higher LDL and lower serum 25(OH)D levels in Chinese patients. The variation in rs2228750 reduced the stability of VDR and changed the binding energy of its ligand, which may be the underlying molecular mechanism for variant associated dyslipidemia. Our findings provide new insights into the prevention and treatment of dyslipidemia. Nevertheless, further studies are required to verify these results in different ethnic groups. Finally, additional functional investigations of the *VDR* gene are needed to confirm the present findings.

## Additional file

Additional file 1: **Table S1.** Clinical characteristics of cases and controls (stage I). **Table S2.** Clinical characteristics of cases and controls (stage II). **Table S3.** Analysis of rs2228570 association with lipid levels. **Table S4.** Analysis of the association of rs2228570 and serum 25(OH)D levels in cases with high LDL. **Table S5.** Changes in VDR stability. **Table S6.** Changes in binding energy of VDR. (DOC 241 kb)

## Abbreviations

LDL-C: Low density lipoprotein cholesterol
SNPs: Single nucleotide polymorphisms
TC: Total cholesterol
VDR: Vitamin D receptor
