# Supplementary material for: Vitamin D receptor polymorphism rs2228570 is significantly associated with risk of dyslipidemia and serum LDL levels in Chinese Han population
Source: Lipids Health Dis. 2018 Aug 17;17:193. doi: 10.1186/s12944-018-0819-0 (PMC6098609; doi:10.1186/s12944-018-0819-0)
Supplement: Supplementary file 1 — Table S1. Clinical characteristics of cases and controls (stage I). Table S2. Clinical characteristics of cases and controls (stage II). Table S3. Analysis of rs2228570 association with lipid levels. Table S4. Analysis of the association of rs2228570 and serum 25(OH)D levels in cases with high LDL. Table S5. Changes in VDR stability. Table S6. Changes in binding energy of VDR. (DOC 241 kb) [file 12944_2018_819_MOESM1_ESM.doc]

**Table S1. Clinical characteristics of cases and controls (stage I).**

| Clinical characteristics | Case  (n = 877) | Control  (n = 1882) | Χ^2^/*t* | ***P*** |
| --- | --- | --- | --- | --- |
| Gender (male/female) | 436/441 | 926/956 | 0.063 | 0.802 |
| Age (years) | 58 ± 11 | 58 ± 11 | -0.77 | 0.938 |
| BMI (kg·m^2^) | 24.82 ± 3.14 | 23.59 ± 3.2 | 12.79 | <0.001 |
| SBP (mmHg) | 121 ± 11 | 120 ± 12 | 2.35 | 0.125 |
| DBP (mmHg) | 77 ± 7 | 77 ± 7 | 0.522 | 0.47 |
| GLU (mmol/L) | 5.19 ± 0.88 | 5.13 ± 0.68 | -1.71 | 0.086 |
| TC (mmol/L) | 5.27 ± 1.31 | 4.74 ± 0.78 | -10.95 | <0.001 |
| TG (mmol/L) | 2.33 ± 1.61 | 1.15 ± 2.18 | -15.84 | <0.001 |
| LDL-C (mmol/L) | 3.05 ± 1.04 | 2.73 ± 0.59 | -8.519 | <0.001 |
| HDL-C (mmol/L) | 1.21 ± 0.36 | 1.43 ± 0.28 | 16.31 | <0.001 |

Low-density lipoprotein cholesterol (LDL-C), high-density lipoprotein cholesterol (HDL-C), total cholesterol (TC), triglyceride (TG), fasting blood glucose (GLU), body mass index (BMI), systolic blood pressure (SBP), and diastolic blood pressure (DBP)

**Table S2. Clinical characteristics of cases and controls (stage II).**

| Clinical characteristics | Case  (n = 1679) | Control  (n = 3124) | Χ^2^/*t* | ***P*** |
| --- | --- | --- | --- | --- |
| Gender (male/female) | 834/ 845 | 1515/1609 | 0.605 | 0.437 |
| Age (years) | 59.04 ± 11.5 | 59.18 ± 11.3 | 0.407 | 0.684 |
| BMI (kg·m^2^) | 24.82 ± 3.14 | 23.59 ± 3.2 | 12.79 | <0.001 |
| SBP (mmHg) | 130 ± 16 | 128 ± 17 | 4.81 | <0.001 |
| DBP (mmHg) | 83 ± 9 | 81 ± 9 | 6.41 | <0.001 |
| GLU (mmol/L) | 5.27 ± 0.86 | 5.18 ± 0.72 | 3.73 | <0.001 |
| TC (mmol/L) | 5.26 ± 1.28 | 4.74 ± 0.78 | 17.58 | <0.001 |
| TG (mmol/L) | 2.48 ± 1.71 | 1.17 ± 1.72 | 25.13 | <0.001 |
| LDL-C (mmol/L) | 3.04 ± 1.03 | 2.72 ± 0.59 | 13.54 | <0.001 |
| HDL-C (mmol/L) | 1.21 ± 0.35 | 1.44 ± 0.28 | 23.91 | <0.001 |
| Hypertension (%) | 47.7% | 39.7% | 28.66 | <0.001 |

Low-density lipoprotein cholesterol (LDL-C), high-density lipoprotein cholesterol (HDL-C),

total cholesterol (TC), triglyceride (TG), fasting blood glucose (GLU), body mass index (BMI), systolic blood pressure (SBP), and diastolic blood pressure (DBP)

**Table S3. Analysis of rs2228570 association with lipid levels.**

| Genotype | lipid level | | |  |
| --- | --- | --- | --- | --- |
|  | TG ≥ 2.26mmol/L | TC ≥ 6.22  mmol/L | LDL ≥ 4.41  mmol/L | HDL < 1.04  mmol/L |
| CC | 3.47 ± 1.78  (n = 261) | 6.82 ± 0.57  (n = 170) | 4.66 ± 0.44  (n = 111) | 0.92 ± 0.11  (n = 236) |
| TC | 3.66 ± 1.93  (n = 404) | 6.83 ± 0.67  (n = 223) | 4.67 ± 0.47  (n = 139) | 0.92 ± 0.09  (n = 358) |
| TT | 3.55 ± 1.52  (n = 174) | 6.87 ± 0.68  (n = 73) | 4.93 ± 0.75  (n = 42) | 0.91 ± 0.1  (n = 143) |
|  | *P* = 0.395 | *P* = 0.841 | ***P*** **= 0.01** | *P* = 0.798 |

**Table S4. Analysis of the association of rs2228570 and serum 25(OH)D levels in cases with high LDL.**

|  |  |  | | | Genotype | | |  |
| --- | --- | --- | --- | --- | --- | --- | --- | --- |
|  |  |  |  |  | CC  N = 111 | TC  N = 139 | TT  N = 42 | |
|  | 25(OH)D (ng/mL) | |  | | 36.76 ± 8.10 | 26.24 ± 4.16 | 18.43 ± 5.04 | |
|  | F | |  | | 194.71 |  |  | |
|  | P | |  | | <0.001 |  |  | |
|  | | | |  | |  |  | |

**Table S**5. Changes in VDR stability.

| Calculate Mutation  (Stability) | Energy Mutations | Residue | A:MET1 |
| --- | --- | --- | --- |
|  |  | Mutated To | THR |
|  |  | Mutation Energy (kcal/mol) | 0.651965 |
|  |  | Effect | destabilizing |

**Table S6. Changes in binding energy of VDR.**

|  | Binding Energy  (kcal/mol) | Ligand Energ  y (kcal/mol) | Protein Energy  (kcal/mol) | Complex Energy  (kcal/mol) | Entropic Energy  (kcal/mol) |
| --- | --- | --- | --- | --- | --- |
| WT | 15,669.10786 | 212.73528 | －16527 | －645.30725 | 22.39100 |
| MUT | 323.48569 | 212.73528 | －12547 | －12,010.97922 | 22.39070 |
